# Dysregulation of store-operated calcium entry in fibroblast lines from adult and juvenile-onset Huntington’s disease patients

**DOI:** 10.1007/s43440-025-00820-8

**Published:** 2026-01-20

**Authors:** Samuel Oluwafemi Egbuwalo, Ewelina Latoszek, Hana Hansíková, Jiří Klempíř, Alžbeta Mühlbäck, Georg Bernhard Landwehrmeyer, Jacek Kuźnicki, Magdalena Czeredys

**Affiliations:** 1 Laboratory of Neurodegeneration, International Institute of Molecular and Cell Biology in Warsaw, Warsaw, Poland; 2 Laboratory for Study of Mitochondrial Disorders, Department of Pediatrics and Inherited Metabolic Disorders, First Faculty of Medicine, Charles University and General University Hospital, Prague, Czech Republic; 3 Department of Neurology, Center of Clinical Neuroscience, First Faculty of Medicine, Charles University and General University Hospital, Prague, Czech Republic; 4 Isar-Amper-Klinikum, Huntington-Zentrum-Süd, Klinik Taufkirchen, Taufkirchen, Germany; 5 Department of Neurology, Ulm University, Ulm, Germany; 6 Department of Stem Cell Bioengineering, Mossakowski Medical Research Institute, Polish Academy of Sciences, Pawińskiego 5, Warsaw, 02-106 Poland

**Keywords:** Huntington’s disease, SOCE, Fibroblasts, Huntingtin

## Abstract

**Background:**

The pathology of Huntington’s disease (HD) is marked by the aggregation of mutant huntingtin protein (mHTT), which results from expanded polyglutamine (polyQ) residues encoded by CAG repeats in the *HTT* gene. These repeats are differentially elongated in adult- and juvenile-onset HD. In striatal neurons, the mHTT disrupts cellular mechanisms such as store-operated calcium entry (SOCE), a process in which endoplasmic reticulum Ca²⁺ depletion triggers extracellular Ca²⁺ influx; however, this process can also be affected in peripheral cells. The aim of this study was to evaluate SOCE in fibroblasts derived from both HD onset patients and age-related controls.

**Methods:**

We conducted SOCE analysis in dermal fibroblasts from 12 HD patients (including adult- and juvenile-onset subtypes) and age-related healthy controls using Fura-2 AM ratiometric imaging paired with EGTA-based extracellular calcium chelation protocols. To evaluate SOCE response, we administered two SOC channel inhibitors, 6-bromo-N-(2-phenylethyl)-2,3,4,9-tetrahydro-1 H-carbazol-1-amine hydrochloride (C_20_H_22_BrClN_2_) and EVP4593, in premanifest HD fibroblasts.

**Results:**

In healthy human fibroblast lines, a decline in SOCE was observed between juvenile and adult individuals. In fibroblast lines from adult-onset HD patients (premanifest, early manifest, and manifest stages), we observed increased SOC channel activity. Conversely, juvenile-onset HD fibroblast lines exhibited reduced SOC channel activity compared to controls. Notably, SOCE dysregulation was independent of CAG repeat length in HD lines. Both SOC channel inhibitors attenuated SOCE in adult-onset HD lines.

**Conclusion:**

The mHTT upregulates SOCE in adult-onset HD fibroblasts and downregulates it in juvenile-onset HD fibroblast lines; however, SOCE levels do not correlate with the length of CAG repeats encoding mHTT. Despite opposing trends compared to age-related controls, similar levels of SOCE in both HD-onset fibroblasts were detected. Both C_20_H_22_BrClN_2_ and EVP4593 show potential for stabilizing SOCE in adult-onset HD. These findings suggest that dysregulated SOCE could be investigated as a peripheral target for studying pathological processes potentially associated with Huntington’s disease.

**Graphical Abstract:**

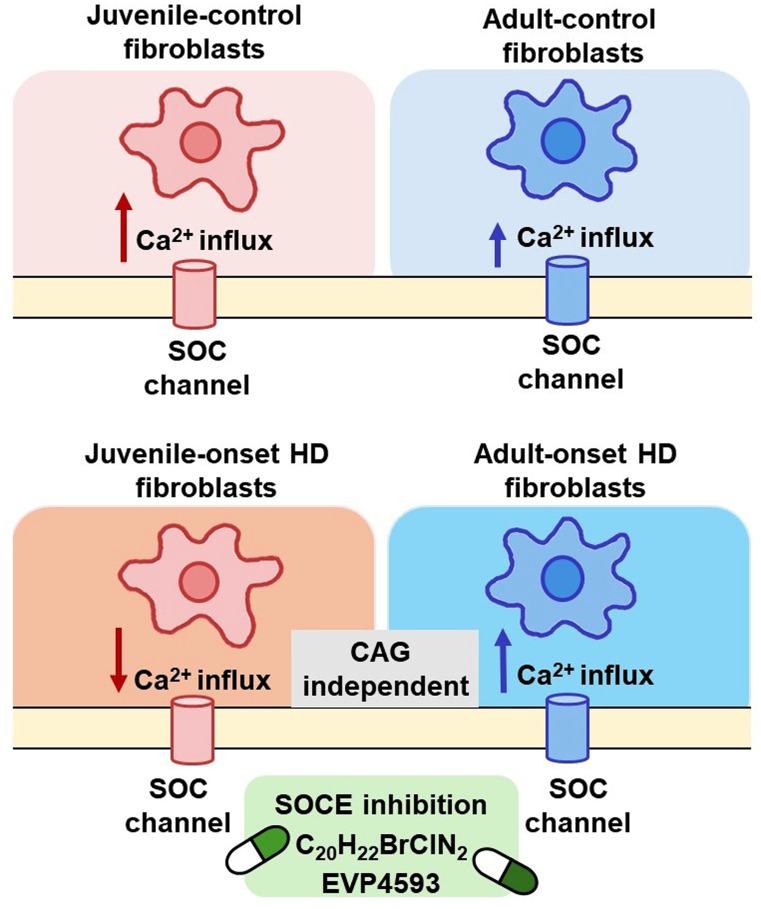

## Introduction

Huntington’s disease (HD) is a progressive neurodegenerative disorder with an autosomal-dominant inheritance, characterized by the aggregation of mutant huntingtin protein (mHTT), which contains an expansion of polyglutamine (polyQ) residues in its N-terminal part [1]. The *mHTT* gene has over 36 CAG repeats and represents a pathological form of HD. In wildtype HTT, the polyQ stretch is under 35 repeats. An increase in CAG length in HD patients correlates with a decrease in their age of onset and earlier neurological symptoms [2]. The vast majority of HD cases carry a canonical CAG repeat region with an uninterrupted CAG repeat followed by a CAACAG allele [3]. Recent findings based on rare HD variants with the loss of a glutamine-encoding CAA allele show that pure CAG repeat length determines the timing of onset, while the polyQ tail seems to be a toxicity driver [3]. Between 40 and 60 CAG repeats in *HTT* cause adult-onset HD at the age of 30–50 years, representing ~ 95% of all HD cases. Clinical manifestations of adult-onset HD include chorea, cognitive decline, and psychiatric disturbances. In contrast, the juvenile form (J-HD) presents before the age of 21 [4] and can occur even in early childhood [5, 6]. J-HD is usually associated with more than 60 CAG repeats and it is caused by anticipation, usually via paternal transmission [5, 6]. The striatum is the earliest and most severely affected brain region in HD, marked by degeneration of medium spiny neurons (MSNs) [7]. Notably, disturbances in Ca^2+^ signaling pathways have been identified in HD models and postmortem brain tissues from HD patients [8–11], suggesting that abnormal Ca²⁺ homeostasis is an early pathological event in HD [12]. mHTT impairs the function of numerous proteins, including transcription factors, and key components of Ca^2+^ homeostasis and signaling pathways [13]. One such pathway, store-operated calcium entry (SOCE), plays a central role in Ca^2+^ signaling in non-excitable cells [14] and it is also active in neurons [15, 16]. Upon activation of inositol 1,4,5-trisphosphate receptors (IP_3_Rs), endoplasmic reticulum (ER) Ca²⁺ is released, leading to Ca^2+^ influx through SOC channels. The ER Ca^2+^ sensors, stromal interaction molecule 1 (STIM1) and stromal interaction molecule 2 (STIM2), detect changes in Ca^2+^ concentrations and undergo conformational changes, forming punctate structures that bring ER membranes in close proximity toward plasmalemma, where SOCE channels - such as Orai or transient receptor potential canonical channels (TRPCs) - are located. This interaction facilitates Ca^2+^ influx entry from the extracellular space through activated members of Orai channels into the cytoplasm and subsequently to the ER [17]. SOCE has been reported to be elevated in several models of HD, such as YAC128 MSNs [10, 11, 18], a cellular HD model [19], and induced pluripotent stem cells (iPSC)-based gamma-aminobutyric acid (GABA)ergic MSNs from HD patient fibroblasts [20–22]. In YAC128 MSNs, an increase in STIM2 expression elevates synaptic SOCE and likely underlies synaptic loss in MSNs [11]. STIM1 expression was unchanged in HD MSNs. The inhibition of TRPC1-dependent SOCE improves synaptic stability [23].

Apart from neuronal cells, HD pathology was recently observed in fibroblast cell lines from HD patients [24]. Mitochondrial structure, mitochondrial fission, and cristae organization were significantly disrupted in adult-onset HD fibroblasts, and elevated apoptosis was detected. Functional alteration of mitochondria confirmed that damage in the energy metabolism in HD may play a role in the pathogenesis of HD in peripheral tissues. Elevated levels of reactive oxygen species (ROS), increased mitochondrial membrane potential, and reduced expression of mitochondrial fusion and fission proteins, along with decreased mitochondrial branching, were observed in fibroblasts from juvenile-onset HD patients; however, cellular viability was preserved by enhanced proteasome activity [25]. Mutant HTT that affects IP_3_R1 [26] and then dysregulates SOCE [27] may play a role in the physical interaction between ER and mitochondria, leading to altered shuttling of Ca^2+^ between the two organelles and in the modulation of the mitochondrial Ca^2+^ uptake [28].

In the present study, we aimed to examine one of the key calcium signaling pathways, SOCE, in dermal fibroblasts derived from both adult- and juvenile-onset HD patients. We report for the first time that SOCE is dysregulated in fibroblast lines from both HD onsets compared to age-related controls, indicating the systemic nature of this pathology. We confirmed that SOCE inhibitors C_20_H_22_BrClN_2_ and EVP4593 have potential for stabilizing SOCE in adult-onset HD fibroblasts. SOCE dysregulation has been found in other neurodegenerative diseases, suggesting similar pathological mechanisms.

## Materials and methods

### Fibroblast cell lines

The HD stage of patients who provided skin biopsies for fibroblast cultures was assessed using neurological tests based on the Unified Huntington’s Disease Rating Scale (UHDRS) [29, 30] and donors were without comorbidities and non-smokers. Control subjects who provided skin biopsies for fibroblast cultures were selected among healthy people. Human dermal fibroblast lines were derived from adult and juvenile HD patients and age-related controls (Table 1). In adult-onset HD, early manifest HD (EM-HD), premanifest HD (PM-HD), and manifest HD (M-HD) fibroblast lines were obtained, as well as corresponding adult control (A-K) lines. Juvenile HD (J-HD) and corresponding juvenile control lines (J-K) were also derived. All cells were obtained from HD patients and controls upon approval of the Ethics Committee of the General University Hospital, Prague (decision no. 1,773,119 S-tV from 17.10.2019) and relevant informed consents were signed by the participants of the study. The GM09197 and GM05539 cell lines were obtained through secondary distribution with the approval of the Coriell Institute for Medical Research. Each HD and control group included at least three lines from three different individuals. All patient-derived fibroblast lines were anonymized.

**Table 1:** HD and control fibroblast lines

Fibroblast lines HD onsets and controls	Fibroblast line name	CAG length	Sex	Age at sampling	Age of onset
Juvenile HD (J-HD)	JHD-V	26/54	F	30	22
	JHD-GM05539	22/101	M	10	2
	JHD-GM09197	21/151	M	6	Not specified
Juvenile control (J-K)	JK-N	23/23	F	29	Not applicable
	JK-KO	CAG*	M	30	Not applicable
	JK-KU	CAG*	M	21	Not applicable
Premanifest HD (PM-HD)	PM-F	15/41	M	48	Not applicable
	PM-S	17/49	F	24	Not applicable
	PM-H	17/41	F	47	Not applicable
Early manifest (EM-HD)	EM-L	12/42	F	59	58
	EM-K	25/53	F	34	32
	EM-H	17/43	F	45	44
Manifest HD (M-HD)	M-KOP	15/45	F	39	36
	M-T	17/47	F	43	40
	M-KER	15/43	F	51	46
Adult control (A-K)	K-PIC	19/19	M	29	Not applicable
	K-BA	23/23	M	36	Not applicable
	K-BO	CAG*	M	43	Not applicable
	K-PIN	CAG*	F	50	Not applicable

*CAG length not analyzed by sequencing; mean and SD for age at sampling for J-HD (15.33 ± 10.59); J-K (26.67 ± 4.16); PM-HD (39.67 ± 12.56); EM-HD (46.00 ± 12.77), M-HD (44.33 ± 7.00) and A-K (39.5 ± 9.04); mean and SD for HD onset: EM-HD (44.67 ± 10.62); M-HD (40.67 ± 4.11). Anonymized patient-derived fibroblast lines from both sexes F and M: JHD-V, JHD-GM05539, JHD-GM09197, JK-N, JK-KO, JK-KU, PM-F, PM-S, PM-H, EM-L, EM-K, EM-H, M-KOP, M-T, M-KER, K-PIC, K-BA, K-BO and K-PIN. Fibroblast lines represent different HD onsets: J-HD, PM-HD, EM-HD, M-HD and control groups: J-K and A-K. Abbreviations: A-K: adult control; EM-HD: early manifest HD; F: female; J-HD: juvenile HD; J-K: juvenile control; M: male; M-HD: manifest HD; PM-HD: premanifest HD; SD: standard deviation

Fibroblast lines were cultured in DMEM with high glucose (Thermo Fisher Scientific, cat. no. D6429), 1% P/S (Gibco cat. no. 15140122), and 10% FBS (Sigma cat. no. F7524) and maintained at 37 °C with 5% CO_2_. The medium was changed every 3 days. Cells were passaged after reaching 90–100% confluency. Cells were rinsed with 1x PBS and detached with 0.25% Trypsin-EDTA solution (Thermo Fisher Scientific, cat. no. 25200-056) heated to 37 °C. Fibroblasts were banked in heat-inactivated FBS and 10% dimethyl sulfoxide (DMSO) (BioShop cat. no. DMS666.50) and frozen in liquid nitrogen. For calcium imaging, about 14,000 fibroblast cells were seeded per well of Corning™ Culture Slide (LabTek) covered with 1% gelatin (Sigma cat. no. G9391) with 500µL of fibroblast medium.

### Single-cell Ca^2+^ measurements

**Fig. 1:**
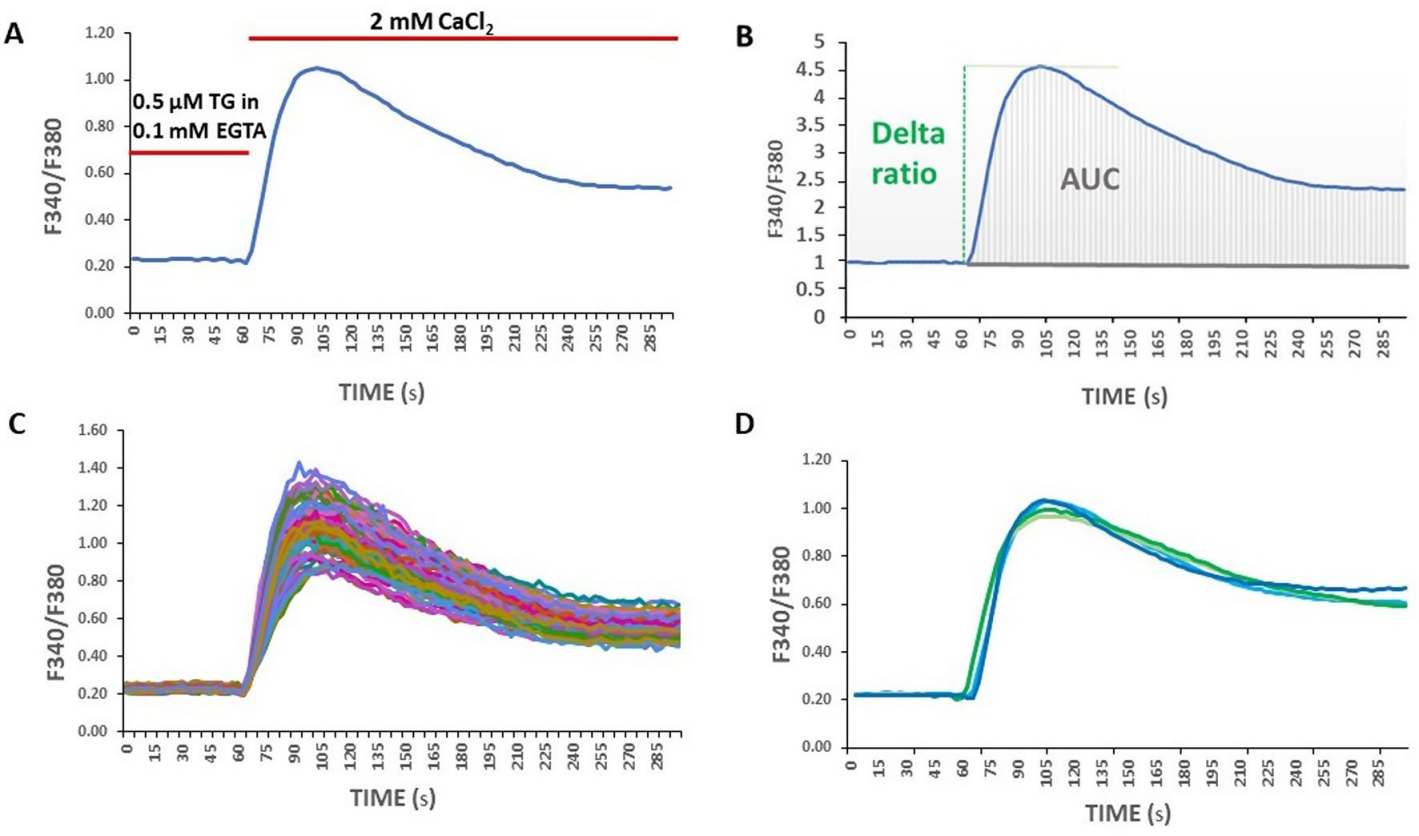
SOCE measurement in fibroblast lines. (**A**) Protocol used to induce SOCE at the 8-well system using juvenile age-related control fibroblast lines shown as example. Fibroblast lines were loaded with the Ca^2+^ probe Fura-2 AM (2 µM). Cells were treated with 0.1 mM EGTA. Ca^2+^ release from the endoplasmic reticulum was induced during 20 min by adding 0.5 µM of TG in 0.1 mM EGTA. Calcium measurements were initiated during the final minute of the TG treatment, followed by re-addition of 2 mM CaCl_2_ to trigger SOCE which is represented by the peak F340/F380. Non-normalized SOCE traces in (**A**). (**B**) The delta ratio (indicated by the green dashed line) calculated as the difference between the peak F340/F380 ratio after extracellular Ca^2+^ was added and its level immediately before the addition of Ca^2+^ as well as the AUC (area filled with gray marks under the line graph) to measure SOCE in juvenile age-related control fibroblast lines. SOCE traces normalized to 1 in (**B**). (**C**) SOCE response in single juvenile age-related control fibroblasts measured in one well. Non-normalized SOCE traces in (**C**). (**D**) Example of average SOCE measurement from different passages and days of calcium imaging of juvenile age-related control fibroblast lines represented by blue and green lines, respectively. Non-normalized SOCE traces in (**D**). Abbreviations: AUC: area under the curve; EGTA: ethylene glycol-bis(β-aminoethyl ether)-N, N,N′,N′-tetraacetic acid; SOCE: Store-operated calcium entry; TG: thapsigargin

Single-cell Ca^2+^ levels in fibroblasts were recorded using the ratiometric Ca^2+^ indicator Fura-2 acetoxymethyl ester (Fura-2 AM) (Thermo Fisher Scientific, cat. no. F1221). Cells grown on 8-well chamber slides were loaded with 2 µM Fura-2 AM in the presence of 0.5% Pluronic (Sigma, cat. no. P2443) for 20 min at 37 °C in a Ringer Buffer that contained 145 mM NaCl (Sigma, cat. no. S9888), 5 mM KCl (Sigma, cat. no. P3911), 0.75 mM Na_2_HPO_4_ (BioShop cat. no. SPD307), 10 mM glucose (Sigma, cat. no. G7021), 10 mM HEPES (pH 7.4) (BioShop cat. no. HEP001) and 1 mM MgCl_2_ (Sigma, cat. no. M0250) supplemented with 2 mM CaCl_2_ (high-Ca^2+^ medium) (Sigma, cat. no. C5670) and then rinsed and left undisturbed for 15 min at 37 °C to allow for de-esterification. The low-Ca^2+^ medium (Ca^2+^-free solution), containing 0.1 mM EGTA (BioShop cat. no. EGT101.25) in the standard buffer, was then added to the cells for 3 min. To induce SOC channels activity, cells were subjected to the following SOCE protocol: ER Ca^2+^ depletion was induced with 0.5 µM thapsigargin (Alomone, cat. no. T-650) in the presence of 0.1 mM EGTA in the standard buffer for 20 min. Calcium measurements began during the final minute of this incubation period, followed by re-addition of 2 mM CaCl_2_ to trigger SOCE (Fig. 1A). Measurements of intracellular Ca^2+^ levels were performed at 37 °C using an Olympus CellˆR imaging software, an IX81 microscope, a 10x/0.4 UPlanSApo objective (Olympus, Japan), and Hamamatsu EM-CCD C9100-02 camera (Hamamatsu Photonics, Japan). Time-lapse imaging was performed every 5 min with a 1-second acquisition interval to capture dynamic changes. Intracellular Ca^2+^ levels in individual fibroblast cell bodies were expressed as the F340/F380 ratio after subtracting background fluorescence. This ratio represents the emission intensities at 510 nm obtained after excitation at 340 and 380 nm. Data processing was performed using Olympus CellˆR software, Microsoft Excel, and GraphPad Prism.

SOCE in fibroblast lines was quantified using both delta ratio and area under the curve (AUC) described earlier [18, 27]. Delta ratio and AUC measured using the trapezoidal method is presented schematically in (Fig. 1B). No significant variability was observed in control analysis when measuring SOCE in single cells in one well (Fig. 1C) or when the average measurement from different fibroblast passages was performed (Fig. 1D).

### Pharmacological treatments

6-bromo-N-(2-phenylethyl)-2,3,4,9-tetrahydro-1 H-carbazol-1-amine hydrochloride (C_20_H_22_BrClN_2)_ [ChemBridge, ID 5265927] was applied to PM-HD fibroblast cultures for 5 min before single-cell Ca^2+^ measurements at a concentration of 10 µM in 0.02% DMSO in HBSS buffer. Herein, 6-bromo-N-(2-phenylethyl)-2,3,4,9-tetrahydro-1 H-carbazol-1-amine hydrochloride will be referred to as tetrahydrocarbazole or C_20_H_22_BrClN_2_. EVP4593 (Sellck Chemicals, cat. no. S4902) at 1 µM in 0.02% DMSO in HBSS buffer was applied to PM-HD fibroblast cultures for 1 h prior to calcium imaging. As a control, PM-HD fibroblast cultures were treated before single-cell Ca^2+^ measurements with 0.02% DMSO for 5 min–1 h, respectively.

### Statistical analysis

**Fig. 2:**
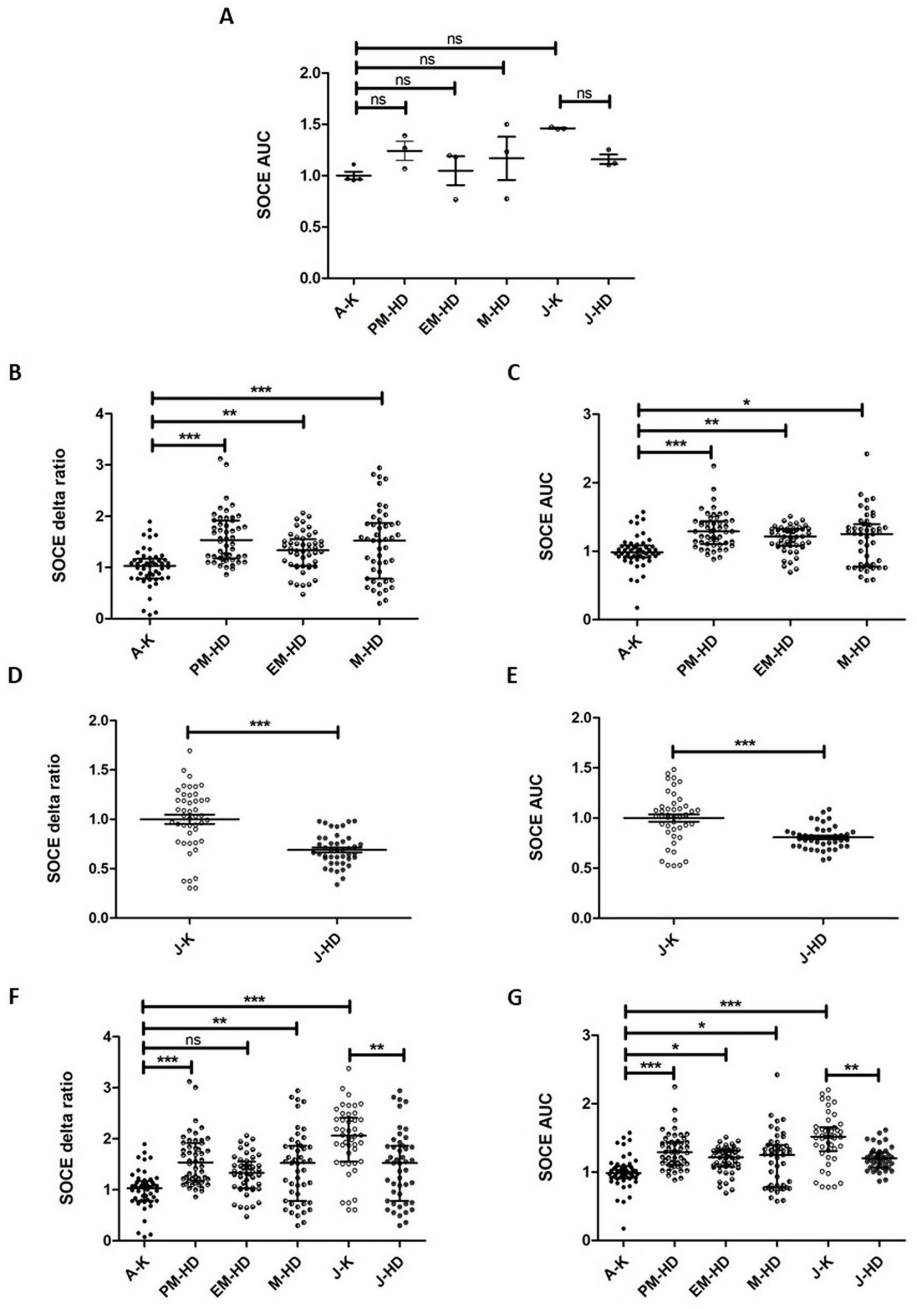
SOCE measurements in fibroblast lines from adult- and juvenile-onset HD compared to age-related controls. Fibroblast lines were loaded with the Ca^2+^ probe Fura-2 AM (2 µM). Cells were treated with 0.1 mM EGTA. Ca^2+^ release from the endoplasmic reticulum was induced by 0.5 µM of thapsigargin in 0.1 mM EGTA. Next, 2 mM of Ca^2+^ was added to induce SOCE. (**A**) SOCE measured as AUC. PM-HD, EM-HD, M-HD, J-HD vs. age-related controls (A-K, J-K) non-significant (ns). Individual data points represent mean from three individuals per group (*N* = 3) except for (*N* = 4) for A-K. The results are shown as mean ± SEM. One-way ANOVA followed by Tukey’s post hoc test. (**B**-**C**) SOCE measured as delta ratio (**B**) or AUC (**C**) was increased in fibroblast lines from PM-HD, EM-HD, and M-HD patients compared to A-K. The results are shown as medians and IQR. Kruskal-Wallis test followed by Dunn’s post hoc test (***, *p* < 0.001; **, *p* < 0.01; *, *p* < 0.05). (**D-E**) SOCE measured as delta ratio (**D**) or AUC (**E**) was decreased in fibroblast lines from J-HD compared to J-K patients. The results are shown as mean ± SEM; Student’s unpaired t-test (***, *p* < 0.001). (**F**-**G**) SOCE measured as delta ratio (**F**) or AUC (**G**) was increased in fibroblast lines from PM-HD and M-HD patients compared to A-K, while it was decreased in J-HD compared to J-K. SOCE measured as AUC (**G**) was increased in EM-HD patients compared to A-K, while it was non-significant when measured as delta ratio (**F**). Additionally, SOCE, measured as the delta ratio (**F**) and AUC (**G**), was decreased in A-K patients compared to J-K. The results are shown as medians and IQR. Kruskal-Wallis test followed by Dunn’s post hoc test (***, *p* < 0.001; **, *p* < 0.01; *, *p* < 0.05, ns, non-significant). Individual data points in Figures (**B**-**G**) correspond to the number of analyzed wells (PM-HD: 50; EM-HD: 48; M-HD: 47; A-K: 49; J-K: 46 and J-HD: 47 for delta ratio and PM-HD: 50; EM-HD: 47; M-HD: 47; A-K: 49; J-K: 46 and J-HD: 43 for AUC), which are technical replicates from at least three patient or control fibroblast lines per group. In each well, approximately 50 ROI were measured, where each ROI corresponds to a single fibroblast cell. The average value from each well was then included in the statistical analysis. Abbreviations: A-K: adult age-related controls; AUC: area under the curve; EGTA: ethylene glycol-bis(β-aminoethyl ether)-N, N,N′,N′-tetraacetic acid; EM-HD: early manifest HD patients; HD: Huntington’s disease; IQR: interquartile ranges; J-HD: juvenile-onset HD patients; J-K: juvenile age-related control; M-HD: manifest-HD patients; PM-HD: premanifest HD patients; SEM: standard error of the mean; SOCE: Store-operated calcium entry; ROI: regions of interest

Data was examined for normal distribution using Kolmogorov Smirnov, D’Agostino and Pearson omnibus and Shapiro-Wilk normality tests. The statistical analysis for parametric data was performed using Student’s unpaired t-test (Figs. 2D-E, 4C and 6C), or a one-way ANOVA followed by Tukey’s post hoc test (Fig. 2A). For non-normally distributed data, the Kruskal-Wallis test (Fig. 2B-C and F-G) or Mann-Whitney U test (4D, 5 A-B, 6 A-B and D) were employed. Dunn’s multiple comparison test was employed to assess significant differences among experimental variants. Correlation analysis in Fig. 4A-B was performed using Pearson’s correlation coefficient. Values of *p* < 0.05 were considered statistically significant while *p* > 0.05 were used to compare the level of significance to that of the control. GraphPad Prism 5.00.288 was used for statistical analysis. In Figs. 2B-G and 4C-D, and Figs. 5 and 6, the statistical significance was assessed using technical replicates of SOCE measurements from independent wells derived from at least three fibroblast lines per experimental group. In Figs. 2A and 4A-B, the statistical significance was evaluated using means calculated from individual patient-derived lines, with at least three lines per group.

## Results

### Dysregulation of SOCE in HD fibroblast lines

To assess whether SOCE is disrupted in peripheral cells of HD patients, we measured thapsigargin-induced Ca²⁺ influx in human dermal fibroblast lines derived from both adult- and juvenile-onset HD patients and appropriate age-related controls (Table 1).

Each examined group included at least three patient-derived fibroblast lines. When the means of SOCE measurements from these fibroblast lines, obtained from adult- and juvenile-onset HD patients, were compared to those from age-related control lines by using one-way ANOVA (Fig. 2A), no statistically significant differences were observed between these groups for AUC analysis (F_5,13_ = 2.385; *p* < 0.0961). However, a trend of SOCE changes was also observed in Fig. 2A between different HD onsets and age-matched controls. Due to the limited number of fibroblast lines available for our study, SOCE in these lines was quantified in the following steps using both the delta ratio and AUC from individual wells; representing technical replicates obtained on the same or different experimental days (Figs. 2B-G, 4C-D, 5 and 6).

A pronounced upregulation of SOCE was observed in fibroblasts from adult-onset HD patients. Specifically, the delta ratio analyzed with the Kruskal-Wallis test revealed a significant difference in SOCE between premanifest HD fibroblasts, early manifest HD fibroblasts, and manifest HD fibroblasts compared to adult control cells (Fig. 2B), with statistical significance H = 33.54 (N_A−K_ = 49, N_PM−HD_ = 50, N_EM−HD_ = 48, N_M−HD_ = 47; *p* < 0.0001). Dunn’s test was used post hoc and demonstrated a significant increase between all analyzed HD variants compared to the adult control group (Fig. 2B). The AUC analysis showed corresponding increases of calcium entry for premanifest HD, early manifest HD, and manifest HD fibroblasts, respectively, compared to adult control cells (Fig. 2C), with statistical significance H = 29.22 (N_A−K_ = 49, N_PM−HD_ = 50, N_EM−HD_ = 48, N_M−HD_ = 47; *p* < 0.0001). Post hoc analyses demonstrated a significant increase in the analyzed HD groups compared to adult controls (Fig. 2C).

Application of the Student’s unpaired t-test exhibited a significant downregulation of SOCE delta ratio in fibroblasts from juvenile-onset HD patients compared to juvenile controls (t_87_ = 5.720; *p* < 0.0001) (Fig. 2D). Similarly, SOCE measured as AUC revealed a significant downregulation in juvenile HD fibroblasts compared to juvenile controls (t_87_ = 4.699; *p* < 0.0001) (Fig. 2E).

**Fig. 3:**
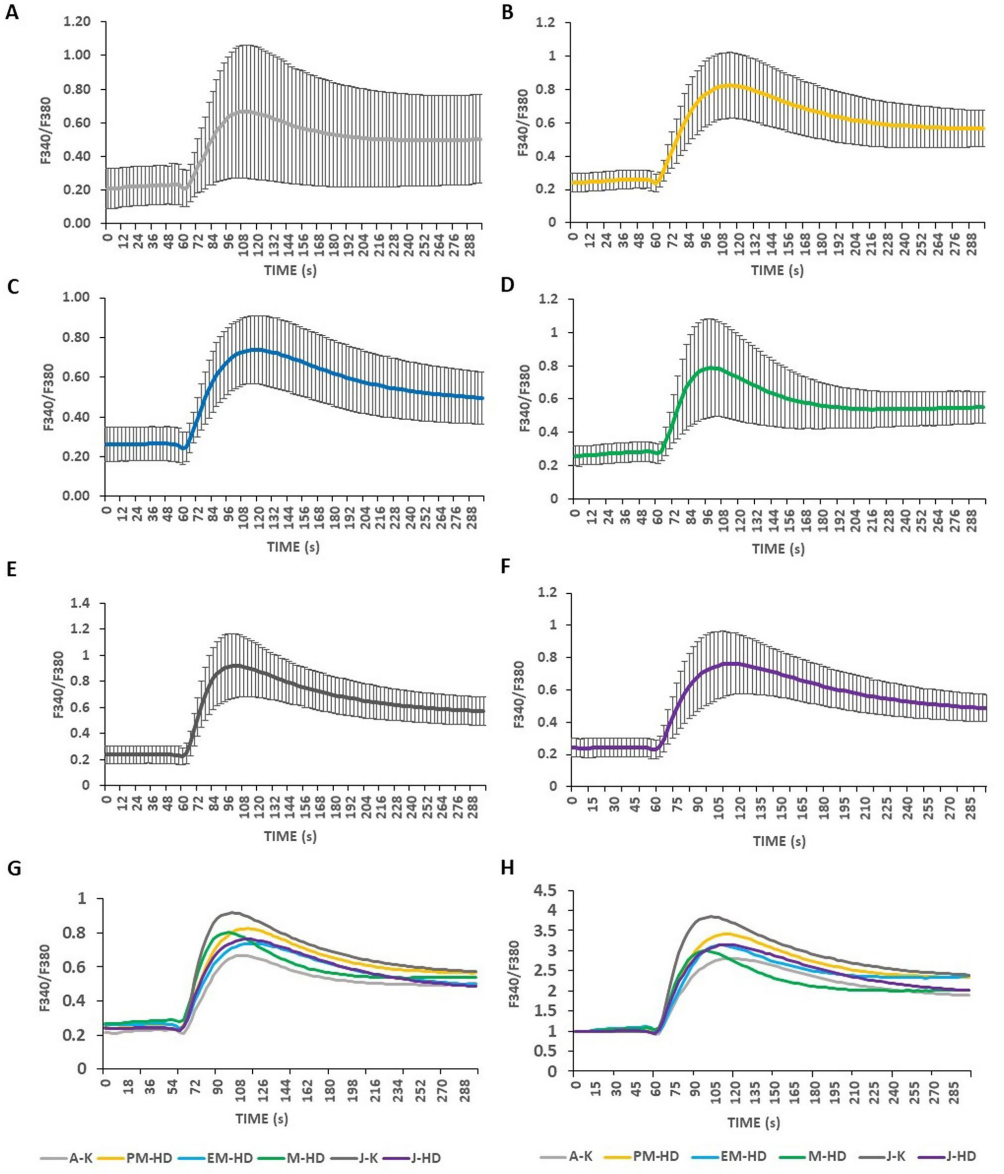
SOCE response in adult- and juvenile-onset HD fibroblast lines and age-related controls. Fibroblast lines were loaded with the Ca^2+^ probe Fura-2 AM (2 µM). Cells were treated with 0.1 mM EGTA. Ca^2+^ release from the endoplasmic reticulum was induced by 0.5 µM of thapsigargin in 0.1 mM EGTA. Next, 2 mM of Ca^2+^ was added to induce SOCE. In Figures (**A**-**H**) SOCE was calculated as the average of wells with fibroblast lines from each experimental variant: (**A**) A-K, (**B**) PM-HD, (**C**) EM-HD, (**D**) M-HD, (**E**) J-K and (**F**) J-HD. Non-normalized (**A**-**G**) and normalized to 1 (**H**) SOCE traces. Abbreviations: A-K: adult age-related controls; EGTA: ethylene glycol-bis(β-aminoethyl ether)-N, N,N′,N′-tetraacetic acid; EM-HD: early manifest HD patients; HD: Huntington’s disease; J-HD: juvenile-onset HD patients; J-K: juvenile age-related control; M-HD: manifest-HD patients; PM-HD: premanifest HD patients; SOCE: Store-operated calcium entry

When all examined fibroblast lines from adult- and juvenile-onset HD patients were compared to age-related control lines using the Kruskal-Wallis test (Fig. 2F), the SOCE delta ratio revealed significant dysregulation of calcium entry (H = 63.39; N_A−K_ = 49, N_PM−HD_ = 50, N_EM−HD_ = 48, N_M−HD_ = 47, N_J−K_ = 46, N_J−HD_ = 47; *p* < 0.0001). Dunn’s test was used post hoc and demonstrated a significant increase between premanifest HD and manifest HD fibroblast lines compared to adult control cells, while no significant differences were detected when comparing adult control lines to early manifest HD variants (Fig. 2F). In contrast, the SOCE delta ratio was significantly downregulated in juvenile HD fibroblasts as well as in adult control fibroblasts compared to juvenile controls (Fig. 2F). The AUC analysis with the Kruskal-Wallis test (Fig. 2G) showed a significant dysregulation of SOCE (H = 60.85, N_A−K_ = 49, N_PM−HD_ = 50, N_EM−HD_ = 48, N_M−HD_ = 47, N_J−K_ = 46, N_J−HD_ = 43; *p* < 0.0001). Post hoc analyses demonstrated a significant increase between premanifest HD, early manifest HD and manifest HD compared to adult control cells, while the SOCE AUC was downregulated in juvenile HD fibroblasts as well as in adult control fibroblasts compared to juvenile control groups (Fig. 2G). Calcium imaging traces representing SOCE response in adult- and juvenile-onset HD fibroblast lines and age-related controls are presented in (Fig. 3).

**Fig. 4:**
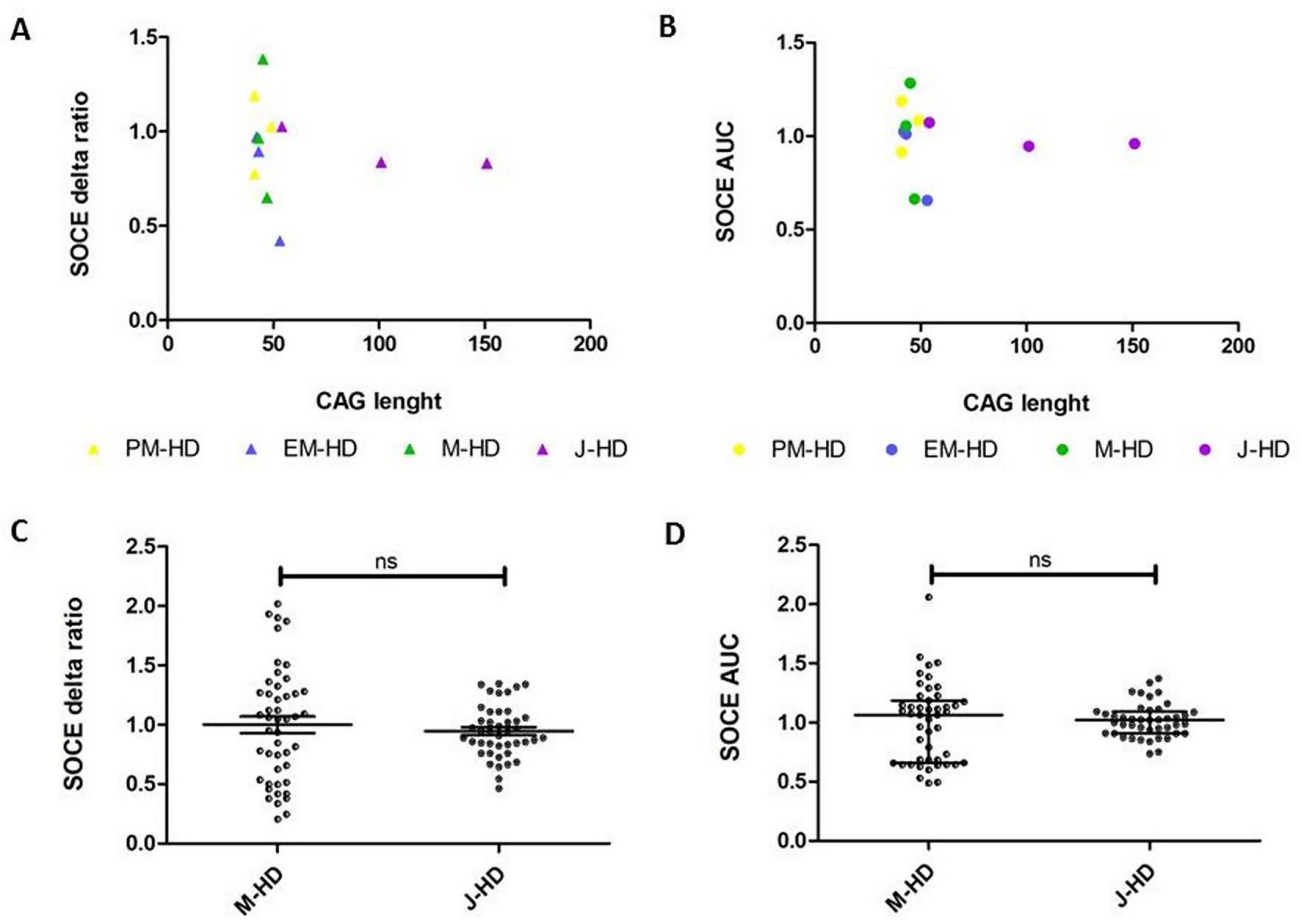
Effect of the CAG length on SOCE in fibroblast lines from juvenile-and adult-onset HD. Fibroblast lines were loaded with the Ca^2+^ probe Fura-2 AM (2 µM). Cells were treated with 0.1 mM EGTA. Ca^2+^ release from the endoplasmic reticulum was induced by 0.5 µM of thapsigargin in 0.1 mM EGTA. Next, 2 mM of Ca^2+^ was added to induce SOCE. (**A**-**B**) The CAG length of the mutant huntingtin has no effect on SOCE measured as delta ratio (**A**) or AUC (**B**) between fibroblasts from J-HD compared to adult-onset HD groups (PM-HD, EM-HD, M-HD). Individual data points in **A-B** represents the average SOCE measurement of each HD patient. Fibroblast HD lines indicated as triangles (**A**) or circles (**B**) are summarized in Table 1 with a corresponding reference to their CAG repeat lengths. In Figures (**A**-**B**) Pearson’s correlation coefficient. (**C**-**D**) The CAG length of the mutant huntingtin has no effect on SOCE measured as delta ratio (**C**) or AUC (**D**) between fibroblasts from J-HD compared to M-HD. In **C**, the results are shown as mean ± SEM; Student’s unpaired t-test (ns; non-significant). In **D**, the results are shown as medians and IQR; Mann-Whitney U test (ns; non-significant). Individual data points in Figures (**C**-**D**) correspond to the number of analyzed wells (M-HD: 47 and J-HD: 43 for both delta ratio and AUC) representing technical replicates from at least three patient-derived fibroblast lines per group. In each well, approximately 50 ROI were measured, where each ROI corresponds to a single fibroblast cell. The average value of each well was included in the statistical analysis. Abbreviations: A-K: adult age-related controls; AUC: area under the curve; EM-HD: early manifest HD patients; HD: Huntington’s disease; IQR: interquartile ranges; J-HD: juvenile-onset HD patients; J-K: juvenile age-related control; M-HD: manifest-HD patients; PM-HD: premanifest HD patients; SEM: standard error of the mean; SOCE: Store-operated calcium entry; ROI: regions of interest

We next examined whether different CAG repeat lengths in the *HTT* gene influenced SOCE in fibroblast lines from both HD onsets. No significant correlation between the number of CAG repeats in the *HTT* gene and the magnitude of SOCE alterations in either juvenile- or adult-onset HD fibroblast lines was detected when analyzing the correlation coefficient (r) and p-value for SOCE measured as delta ratio [*r* = 0.03541, *p* = -0.1882 (Fig. 4A)] or AUC [*r* = 0.04034, *p* = -0.2008 (Fig. 4B)] in relation to CAG length. Moreover, using the Student’s unpaired t-test, no significant differences in SOCE were observed between juvenile-onset HD and manifest HD fibroblast lines differing in CAG length when analyzing delta ratio (t_88_ = 0.6701; *p* = 0.5046) (Fig. 4C). Similarly, no significant differences in SOCE analyzed as AUC using the Mann-Whitney U test were observed for manifest HD compared to juvenile HD fibroblasts (U = 1007, N_M−HD_ = 47, N_J−HD_ = 43; *p* = 0.9807) (Fig. 4D).

**Fig. 5:**
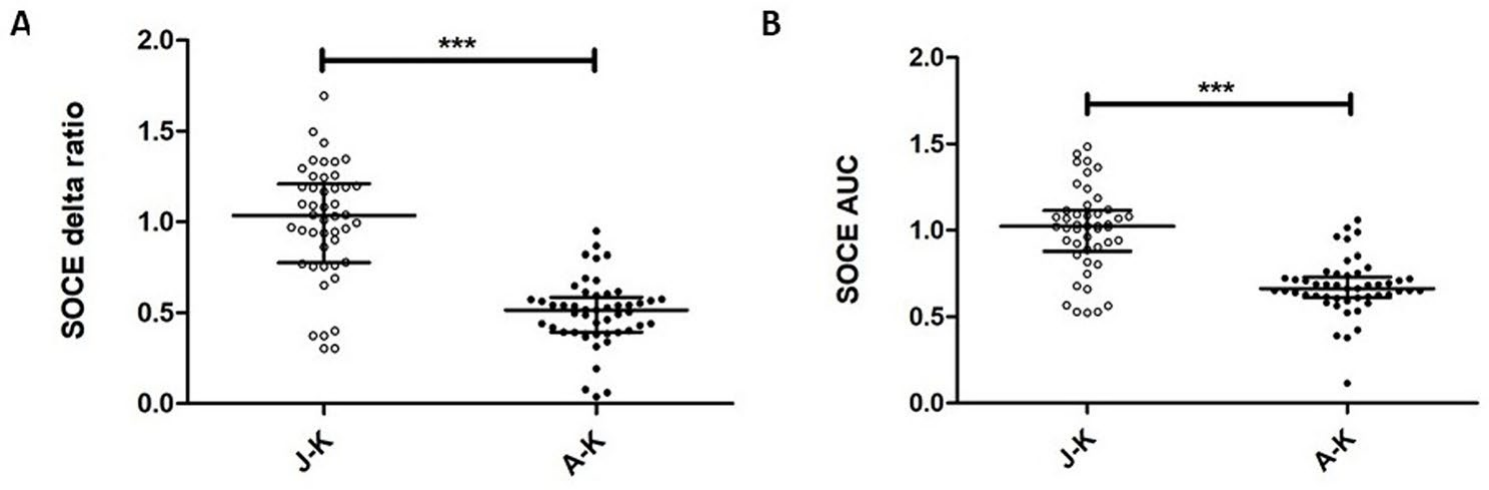
SOCE measurement in fibroblast lines from juvenile and adult controls. Fibroblast lines were loaded with the Ca^2+^ probe Fura-2 AM (2 µM). Cells were treated with 0.1 mM EGTA. Ca^2+^ release from the endoplasmic reticulum was induced by 0.5 µM of thapsigargin in 0.1 mM EGTA. Next, 2 mM of Ca^2+^ was added to induce SOCE. SOCE measured as delta ratio (**A**) and AUC (**B**) was decreased in fibroblast lines from A-K compared to J-K. Individual data points on the graphs correspond to the number of analyzed wells (J-K: 46 and A-K: 49 for both delta ratio and AUC) representing technical replicates from at least three control fibroblast lines per group. In each well, around 50 ROI were measured, where each ROI corresponded to a single fibroblast cell, and then the average value of each well was included in the statistical analysis. The results are shown as medians and IQR; Mann-Whitney U test (***p < 0.0001). Abbreviations: A-K: adult age-related controls; AUC: area under the curve; EGTA: ethylene glycol-bis(β-aminoethyl ether)-N, N,N′,N′-tetraacetic acid; IQR: interquartile ranges; J-K: juvenile age-related control; SEM: standard error of the mean; SOCE: Store-operated calcium entry; ROI: regions of interest

In adult control fibroblasts compared to juvenile control cells, significant differences in SOCE were observed using the Mann-Whitney U test (Fig. 5). The delta ratio analysis showed a decrease in adult control fibroblasts, compared to juvenile control cells (U = 254, N_A−K_ = 49, N_J−K_ = 46; *p* = 0.0001) (Fig. 5A). The AUC analysis showed corresponding decreases in adult control fibroblasts compared to juvenile control cells (U = 340, N_A−K_ = 49, N_J−K_ = 46; *p* = 0.0001) (Fig. 5B).

### Pharmacological inhibition of SOCE restores calcium homeostasis

**Fig. 6:**
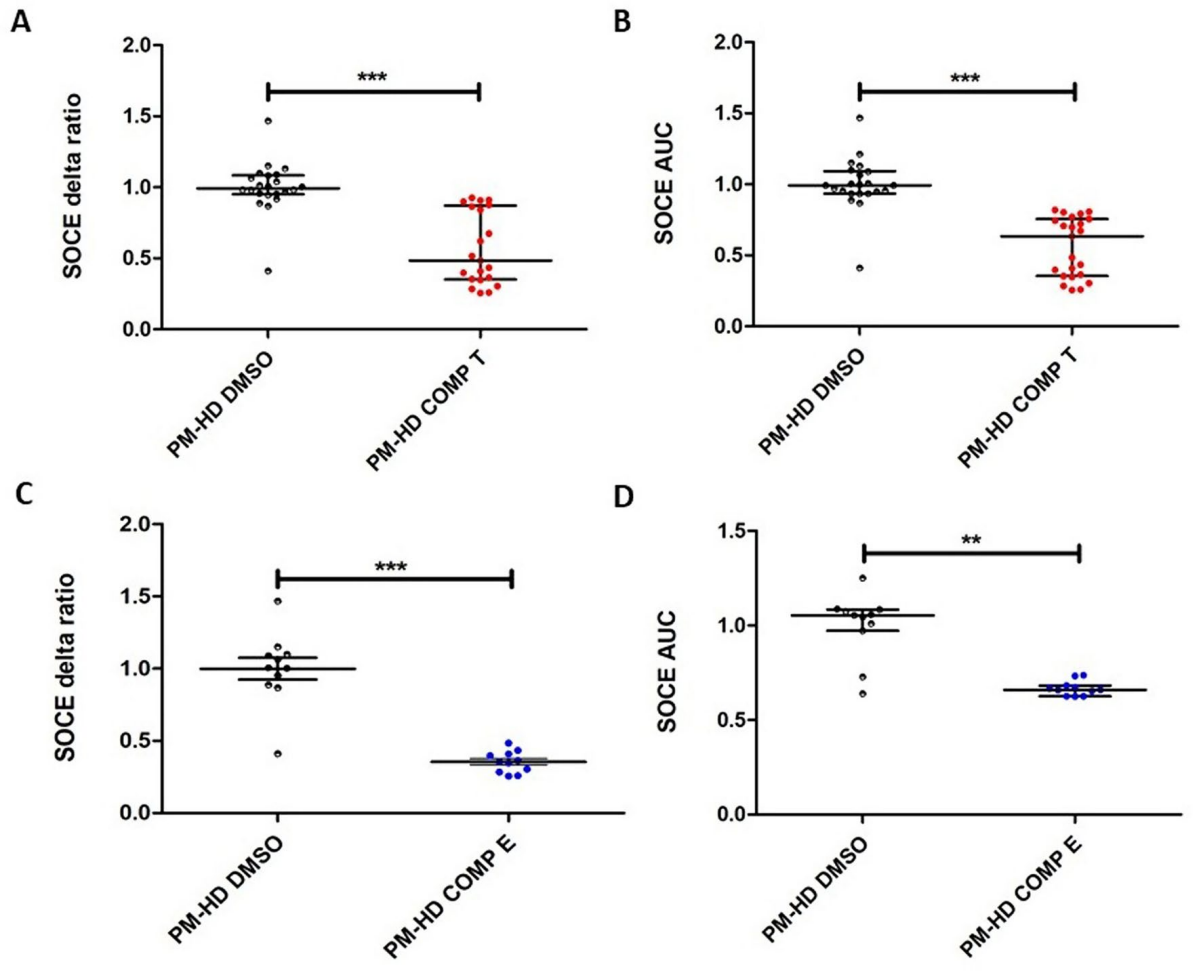
Effect of SOCE inhibitors on premanifest HD fibroblast lines. PM-HD fibroblast lines were incubated for 5 min before single-cell Ca^2+^ measurements with 10 µM tetrahydrocarbazole in 0.02% DMSO indicated as red points (**A**-**B**) or 1h with 1 µM EVP4593 in 0.02% DMSO (blue points) (**C**-**D**) and in 5 min-1h 0.02% DMSO as a control, respectively (black points) (**A**-**D**). Fibroblast lines in **A**-**D** were loaded with the Ca^2+^ probe Fura-2 AM (2 µM). Cells were treated with 0.1 mM EGTA. Ca^2+^ release from the endoplasmic reticulum was induced by 0.5 µM of thapsigargin in 0.1 mM EGTA. Next, 2 mM of Ca^2+^ was added to induce SOCE. (**A**-**B**) Tetrahydrocarbazole attenuate SOCE in premanifest HD fibroblast lines measured as the delta ratio (**A**) or AUC (**B**) in PM-HD fibroblast lines treated with tetrahydrocarbazole (T) compared to PM-HD cells treated with DMSO. Individual data points on the graphs correspond to the number of analyzed wells (PM-HD DMSO: 22 and PM-HD COMP T: 21 for delta ratio and PM-HD DMSO: 23 and PM-HD COMP T: 22 for AUC). (**C**-**D**) EVP4593 attenuate SOCE in premanifest HD fibroblast lines measured as the delta ratio (**C**) or AUC (**D**) in PM-HD fibroblast lines treated with EVP4593 (**E**) compared to PM-HD cells treated with DMSO. Individual data points on the graphs correspond to the number of analyzed wells (PM-HD DMSO: 11 and PM-HD COMP E: 11 for both delta ratio and AUC). In each well in Figures (**A**-**D**), approximately 50 ROI were measured, where each ROI corresponds to a single fibroblast cell. The average value from each well which was technical replicate was then included in the statistical analysis. In Figures (**A**-**B**) the results are shown as medians and IQR; Mann-Whitney U test (***, *p* < 0.0001). In **C**, the results are shown as mean ± SEM; Student’s unpaired t-test and (***, *p* < 0.0001). In **D**, the results are shown as medians and IQR; Mann-Whitney U test (**, *p* < 0.001). Abbreviations: AUC: area under the curve; DMSO: dimethyl sulfoxide; EGTA: ethylene glycol-bis(β-aminoethyl ether)-N, N,N′,N′-tetraacetic acid; HD: Huntington’s disease; IQR: interquartile ranges; PM-HD: premanifest HD patients; PM-HD DMSO: premanifest HD cells treated with DMSO; PM-HD COMP E: premanifest HD cells treated with EVP4593; PM-HD COMP T: premanifest HD cells treated with Tetrahydrocarbazole; SEM: standard error of the mean; SOCE: Store-operated calcium entry; ROI: regions of interest

To test whether pharmacological inhibition could normalize SOCE, we applied the tetrahydrocarbazole derivative C_20_H_22_BrClN_2_ to premanifest HD fibroblast lines. Tetrahydrocarbazoles were previously found to restore SOCE in neuronal HD models [18, 27]. Fibroblasts were pretreated with 10 µM C_20_H_22_BrClN_2_ for 5 min before SOCE measurement. Statistical analysis using the Mann-Whitney U test revealed a significant decrease of SOCE delta ratio in premanifest HD fibroblasts treated with tetrahydrocarbazole compared to premanifest HD cells treated with 0.02% DMSO (U = 22, N_PM−HD COMP T_ = 21, N_PM−HD DMSO_ = 22; *p* < 0.0001) (Fig. 6A). The AUC analysis revealed a significant decrease of SOCE in premanifest HD fibroblasts treated with tetrahydrocarbazole compared to premanifest HD cells treated with 0.02% DMSO (U = 14, N_PM−HD COMP T_ = 23, N_PM−HD DMSO_ = 22; *p* < 0.0001) (Fig. 6B).

Similarly, the SOCE inhibitor EVP4593, which exerts neuroprotective effects in HD models [10, 21], was applied to premanifest HD fibroblast cultures at a concentration of 1 µM for 1 h before measurement. In premanifest fibroblasts treated with EVP4593, a significant decrease of SOCE delta ratio was observed using the Student’s unpaired t-test compared to premanifest HD treated with 0.02% DMSO (t_20_ = 8.103; *p* < 0.0001) (Fig. 6C). The AUC analysis showed significantly reduced SOCE in premanifest HD fibroblasts treated with EVP4593 compared to premanifest HD cells treated with 0.02% DMSO using the Mann-Whitney U test (U = 10, N_PM−HD COMP E_ = 11, N_PM−HD DMSO_ = 11; *p* < 0.001) (Fig. 6D).

## Discussion

Our study broadens the understanding of calcium signaling disturbances in HD by demonstrating that SOCE dysregulation, previously described in neuronal models [10, 18, 21, 27], is also evident in human fibroblasts. This finding underscores the systemic nature of calcium homeostasis disruption in HD, which extends beyond the nervous system.

We observed a substantial upregulation of SOCE measured as delta ratio in fibroblasts derived from adult-onset HD patients including premanifest HD, early manifest HD, and manifest HD lines, respectively, compared to adult age-related controls. Similar results were obtained when SOCE was measured as AUC. These results are consistent with prior studies in MSNs from YAC128 HD mice and iPSC-derived neurons from adult-onset HD with 40–47 CAG repeats, where increased SOCE was linked to elevated IP_3_R1 activity and upregulation of regulatory proteins such as STIM2 and HTT-associated protein-1 isoform A (HAP1A) [10, 20, 27]. Overexpression of STIM2 and HAP1A has been shown to enhance SOCE by promoting ER Ca²⁺ depletion, while silencing these proteins normalizes SOCE and prevents synaptic loss [11, 27, 31]. Comparably, knockdowns of TRPC1, Orai1, or STIM1 in neuronal HD models also restored SOCE to normal levels [23]. Similar mechanisms to those observed in HD MSNs may operate in adult-onset HD fibroblasts, although this idea requires direct experimental validation.

We report for the first time a marked downregulation of SOCE measured as delta ratio and AUC in fibroblasts from juvenile HD patients compared to juvenile age-related controls. This is in contrast to an increased SOCE observed in iPSC-derived MSNs from a juvenile HD patient with 76 CAG repeats [22]. Such a discrepancy may be attributed to differences in cell type, developmental stage, or the presence of additional genetic modifiers in juvenile-onset HD. Our data are supported by the observation that PC12 cells expressing full-length mHTT exhibit downregulated SOCE and reduced levels of associated proteins [27]. Similarly to our results in juvenile-onset HD fibroblasts, iPSC-derived NPCs from bipolar disorder patients exhibit reduced SOCE and also accelerated neurodifferentiation [32], supporting a possible role of SOCE attenuation in the neurodevelopmental pathology of bipolar disorder. Recently, juvenile-onset HD was also considered as neurodevelopmental disorder [33]. Findings in juvenile- and adult-onset HD fibroblasts may suggest that SOCE regulation in HD is highly context-dependent, varying with cell type and possibly with patients’ genetic backgrounds. Conversely to adult-onset HD fibroblasts, the reduction of SOCE in juvenile HD fibroblasts may reflect an adaptive or compensatory response to chronic mHTT-induced stress, or differential regulation of SOCE components during development [33].

Our findings demonstrate that SOCE measured as delta ratio and AUC is significantly decreased in fibroblasts derived from adult healthy donors compared to those from juvenile age-related controls. This observation aligns with previous reports indicating that capacitative calcium entry, a process synonymous with SOCE, is attenuated during both in vivo and in vitro aging of fibroblasts [34]. Interestingly, our data confirm that the CAG repeat length encoding the mutant huntingtin protein does not influence SOCE alterations in either juvenile- or adult-onset HD fibroblasts, confirming previous reports in iPSC-derived MSNs from adult- and juvenile-onset HD patients [22]. This suggests that the presence of mHTT, rather than the precise repeat length, is sufficient to disrupt calcium homeostasis, possibly through gain-of-function mechanisms that affect the expression or function of key calcium regulatory proteins. Our observation that SOCE is stabilized by mHTT at similar levels in both juvenile- and adult-onset HD fibroblasts, despite opposing trends when compared to age-related controls, raises important questions about underlying mechanisms. The mHTT has been shown to interact with a variety of calcium-binding proteins and to alter the expression of genes involved in calcium homeostasis at the transcriptional level [8, 31, 33].

Recent studies in adult- and juvenile-onset HD isogenic human embryonic stem cell lines suggest that mitochondrial dysfunction and oxidative stress in HD cells correlate with the CAG repeat length [35], but not necessarily with SOCE changes, which were detected in this study and by others [22]. We conclude that alterations in calcium signaling are independent of the length of CAG repeats and can be a common part of the pathogenesis of adult and juvenile HD in fibroblasts. Mitochondrial impairments were observed in fibroblast lines from both adult- and juvenile-onset HD patients [24, 25]. Dysregulation of SOCE in fibroblast lines from both HD onset types may affect these downstream pathways. Attenuation of SOCE may correspond with mitochondrial dysfunction observed in juvenile-onset HD, which is compensated by an increased proteasome activity [25], whereas adult-onset HD fibroblasts exhibit elevated SOCE alongside mitochondrial abnormalities [24].

Pharmacological modulation of SOCE in PM-HD fibroblast lines was achieved using known SOCE inhibitors, namely the tetrahydrocarbazole derivative C_20_H_22_BrClN_2_ and EVP4593, previously used in HD MSNs [10, 18]. Both tetrahydrocarbazole and EVP4593 substantially reduced SOCE, measured as delta ratio and AUC, in PM-HD fibroblasts. Inhibition of SOCE by these compounds confirmed the detection of SOCE responses in the studied fibroblast lines. SOCE stabilization using tetrahydrocarbazole in adult-onset HD fibroblasts is consistent with its inhibitory effect, which was observed in MSNs from YAC128 mice and HD MSNs expressing the HAP1A isoform [18, 27]. The mechanism likely involves direct or indirect inhibition of SOCE channels, possibly through post-translational modifications or cytoskeletal stabilization [18]. The beneficial effects of tetrahydrocarbazole compounds on mitochondrial membrane potentials in HD MSNs may also be linked to the stabilization of mitochondrial calcium homeostasis [18]. In addition to the role of C_20_H_22_BrClN_2_ in restoration of SOCE in HD models, it was also beneficial in stabilizing calcium homeostasis in Alzheimer’s disease models [36, 37]. Restoration of SOCE in adult-onset HD fibroblast lines by EVP4593 is consistent with prior work that has demonstrated the neuroprotective effects of this compound in HD models [10, 21, 22, 38]. Notably, EVP4593 appears to target SOCE regulatory proteins, such as STIM, rather than specific channel subunits [19].

Our results reinforce the concept that SOCE dysregulation is a hallmark of HD pathology, present in both neuronal and peripheral cells. The distinct patterns observed between adult- and juvenile-onset HD fibroblasts highlight the complexity of calcium signaling disturbances in HD and suggest that therapeutic strategies may need to be tailored to disease onset and cell type. Importantly, the ability of tetrahydrocarbazole and EVP4593 to normalize SOCE in HD [10, 18] underscores their future potential to restore calcium homeostasis and possibly mitigate mitochondrial dysfunction in HD fibroblasts.

In summary, our findings extend the evidence for SOCE dysregulation in HD to human fibroblasts and reveal divergent patterns between adult- and juvenile-onset cases. The observed elevation of SOCE in premanifest HD fibroblasts may be explored as an early mechanism of pathological processes associated with Huntington’s disease.

### Study limitations

We acknowledge that the results being discussed in the present study are subject to the limited number of primary fibroblasts that were used to conduct the research. Although our statistical analyses were performed following the necessary biological replicates (*N* ≥ 3 for each group), Figs. 2B-G and 4C-D, and Figs. 5 and 6 were based on technical replicates of SOCE measurements from independent wells, obtained on the same or different experimental days from at least three fibroblast lines from HD patients or age-related controls in each group. No statistically significant differences but only a trend was observed when averaging SOCE measurements across experimental groups (Fig. 2A). Therefore, future studies with expanded patient cohorts are needed to determine whether these trends achieve statistical significance and to confirm our data.

## Data Availability

The datasets generated and analyzed during the current study are available from the corresponding author upon reasonable request.

## Abbreviations

A-K: Adult control
AUC: Area under the curve
C_20_H_22_BrClN_2_: 6-bromo-N-(2-phenylethyl)-2,3,4,9-tetrahydro-1 H-carbazol-1-amine hydrochloride
DMSO: Dimethyl sulfoxide
EGTA: Ethylene glycol-bis(β-aminoethyl ether)-N, N,N′,N′-tetraacetic acid
EM-HD: Early manifest HD
ER: Endoplasmic reticulum
GABA: Gamma-aminobutyric acid
HAP1A: HTT-associated protein-1 isoform A
HD: Huntington’s disease
*HTT*: Huntingtin gene
iPSC: Induced pluripotent stem cells
IP3Rs: Inositol 1,4,5-trisphosphate receptors
IQR: Interquartile Range
J-HD: Juvenile HD
J-K: Juvenile control
M-HD: Manifest HD
mHTT: Mutant huntingtin protein
MSNs: Medium spiny neurons
ns: Non-significant
Orai: Channels involved in SOCE
UHDRS: Unified Huntington’s Disease Rating Scale
PM-HD: Premanifest HD
polyQ: Polyglutamine
r: Correlation coefficient
ROI: Regions of interest
ROS: Reactive oxygen species
SEM: Standard error of the mean
SD: Standard deviation
SOCE: Store-operated calcium entry
STIM1: Stromal interaction molecule 1
STIM2: Stromal interaction molecule 2
TG: Thapsigargin
TRPCs: Transient receptor potential canonical channels
